# Exercise, Sleep, and Cognitive Performance in Older Adults: A Structured Review of Mechanisms and Clinical Implications

**DOI:** 10.3390/medsci14050532

**Published:** 2026-08-31

**Authors:** Elton Fernando Ferreira de Souza Vieira, Raphael Lopes Olegário, Cláudia Goulart, Otávio Toledo Nóbrega, Emmanuel Frimpong, Arsenio Paez, Thien Thanh Dang-Vu, Einstein Francisco Camargos

**Affiliations:** 1 Faculty of Medicine, University of Brasília, Brasília 70910-900, Brazil; eltonsouzafef@gmail.com (E.F.F.d.S.V.); contact@rlolegario.com (R.L.O.); cgoulart@unb.br (C.G.); otavionobrega@unb.br (O.T.N.); 2 Faculty of Physical Education, University of Brasília, Brasília 70910-900, Brazil; 3 University Hospital of Brasília, University of Brasília, Brasília 70830-900, Brazil; 4 Department of Health, Kinesiology and Applied Physiology, Concordia University, Montreal, QC H4B 1R6, Canada; emmanuel.frimpong@mail.concordia.ca (E.F.); arsenio.paez@concordia.ca (A.P.); tt.dangvu@concordia.ca (T.T.D.-V.); 5 Centre de Recherche de l’Institut Universitaire de Gériatrie de Montréal (CRIUGM), Montreal, QC H3W 1W5, Canada

**Keywords:** physical exercise, sleep quality, cognitive performance, cognitive aging, older adults, mild cognitive impairment, non-pharmacological intervention

## Abstract

**Background**: Age-related cognitive decline is a major public health challenge, and modifiable lifestyle factors such as physical exercise and sleep have emerged as promising targets for preserving cognitive health. Our review aimed to synthesize evidence on the relationships between physical exercise, sleep, and cognitive performance in adults aged ≥60 years, with particular emphasis on the potential mediating or moderating role of sleep. **Methods**: Electronic searches were conducted in PubMed/MEDLINE, EMBASE, the Cochrane Library, Scopus, Web of Science, and Google Scholar to identify studies published between 2015 and 2025. Eligible studies evaluated structured exercise interventions or habitual physical activity and reported at least one sleep outcome alongside at least one cognitive outcome. **Results**: Twelve studies met the inclusion criteria, comprising randomized controlled trials, non-randomized intervention studies, and observational studies. Moderate-intensity aerobic exercise, multicomponent training, and mind–body interventions were the most frequently investigated exercise modalities. Across studies, exercise was generally associated with improved sleep quality and maintenance or enhancement of cognitive performance. Several studies identified sleep quality or sleep efficiency as potential mediators or moderators of exercise-related cognitive benefits; however, heterogeneity in study designs, interventions, sleep assessments, and cognitive outcomes precluded conclusions regarding causality. **Conclusions**: Current evidence suggests that sleep may represent an important behavioral pathway through which exercise contributes to cognitive health in older adults. Further well-designed randomized controlled trials incorporating objective sleep measures and mechanistic biomarkers are warranted to clarify these relationships.

## 1. Introduction

The rapid growth of the aging population has been accompanied by a marked increase in age-related cognitive decline and dementia, placing a substantial burden on persons, families, and healthcare systems [1]. Mild Cognitive Impairment (MCI) is commonly recognized as a critical stage for intervention because cognitive decline is measurable while functional independence is largely preserved [1,2]. Although pharmacological therapies remain limited in their ability to prevent or substantially delay disease progression, growing attention has focused on modifiable lifestyle factors. Among these, physical exercise has emerged as one of the most promising non-pharmacological strategies for preserving cognitive health.

Sleep disturbances are frequent in later life and often involve reduced sleep efficiency, greater fragmentation, and changes in sleep architecture [3]. Poor sleep quality has been associated with poorer cognitive performance and accelerated cognitive decline [4]. In individuals with MCI, persistent sleep problems have also been associated with structural brain changes, including hippocampal alterations (e.g., hippocampal atrophy, reduced hippocampal volume, and impaired hippocampal connectivity), which may contribute to disease progression [1]. Exercise also appears to positively influence sleep regulation, contributing to better sleep quality and efficiency in aging populations [3,5]. Despite these observations, it remains unclear to what extent improvements in sleep contribute to the cognitive benefits associated with exercise. Addressing this knowledge gap is important for understanding the mechanisms through which exercise may promote cognitive health in older adults.

Growing evidence also supports the cognitive benefits of physical activity in older adults [1,6,7]. In persons with MCI, aerobic exercise has been associated with improvements in global cognition and quality of life, supporting exercise as a promising non-pharmacological strategy to preserve cognitive function [1]. Beyond its direct cognitive benefits, exercise also appears to improve sleep. A recent systematic review and meta-analysis found moderate- to high-quality evidence that exercise improves subjective and objective sleep outcomes in older adults with MCI or Alzheimer’s disease and Alzheimer’s disease-related dementias (AD/ADRD), although important gaps remain regarding potential moderators such as sex, napping habits, and medication use [8].

Exercise may influence cognition through multiple biological and behavioral pathways. Proposed mechanisms include enhanced neuroplasticity, partly mediated by increased brain-derived neurotrophic factor (BDNF), together with improvements in sleep quality. Given the central role of sleep in memory consolidation and cognitive function, improving sleep represents one plausible mechanism through which exercise may promote cognitive health in older adults [8,9].

Brain-derived neurotrophic factor (BDNF) is a neurotrophin that contributes to neuronal survival, synaptic plasticity, learning, and memory. Within the brain, exercise-related increases in BDNF signaling may support hippocampal plasticity, synaptic strengthening, and adaptive responses to cognitive demand. Nevertheless, the extent to which muscle-derived BDNF directly contributes to circulating or cerebral BDNF remains uncertain, and its effects may be predominantly local within skeletal muscle. Sleep may interact with these pathways by supporting synaptic plasticity, memory consolidation, and neural recovery. Therefore, exercise-induced neurotrophic signaling and sleep-dependent restorative processes may represent complementary, rather than independent, contributors to cognitive resilience in older adults [10,11].

Although interventions for MCI have shown mixed efficacy, exercise-based approaches (particularly multicomponent programs) have demonstrated potential to improve both cognitive and physical function while providing a safe non-pharmacological strategy to delay cognitive decline [10]. Proposed mechanisms include increased cerebral blood flow, enhanced neuroplasticity, and greater BDNF availability. Combining aerobics, resistance, balance, and flexibility training may provide broader benefits than single-component interventions [6].

Recent neuroimaging studies suggest that higher levels of physical activity and better sleep are associated with greater medial temporal lobe integrity, particularly within hippocampal regions involved in memory [11]. In addition, physical activity may attenuate the adverse cognitive effects associated with poor sleep, suggesting a potential buffering or compensatory mechanism [4,12].

In this review, mediation and moderation are treated as distinct concepts. Mediation refers to an indirect pathway in which exercise influences sleep, which in turn contributes to a subsequent cognitive outcome. Moderation refers to an interaction in which the magnitude or direction of the association between exercise and cognition differs according to sleep characteristic, such as baseline sleep quality or sleep efficiency. These terms are used only when the included study formally tested the corresponding statistical pathway or interaction.

For terminological consistency, physical activity refers to any bodily movement that increases energy expenditure, including habitual or unstructured activity, whereas physical exercise refers to planned, structured, and repetitive activity undertaken to improve or maintain fitness or health. The term physical exercise is used for intervention programs, and physical activity is retained for observational exposure measures or when referring to the broader behavioral construct.

**Figure 1:**
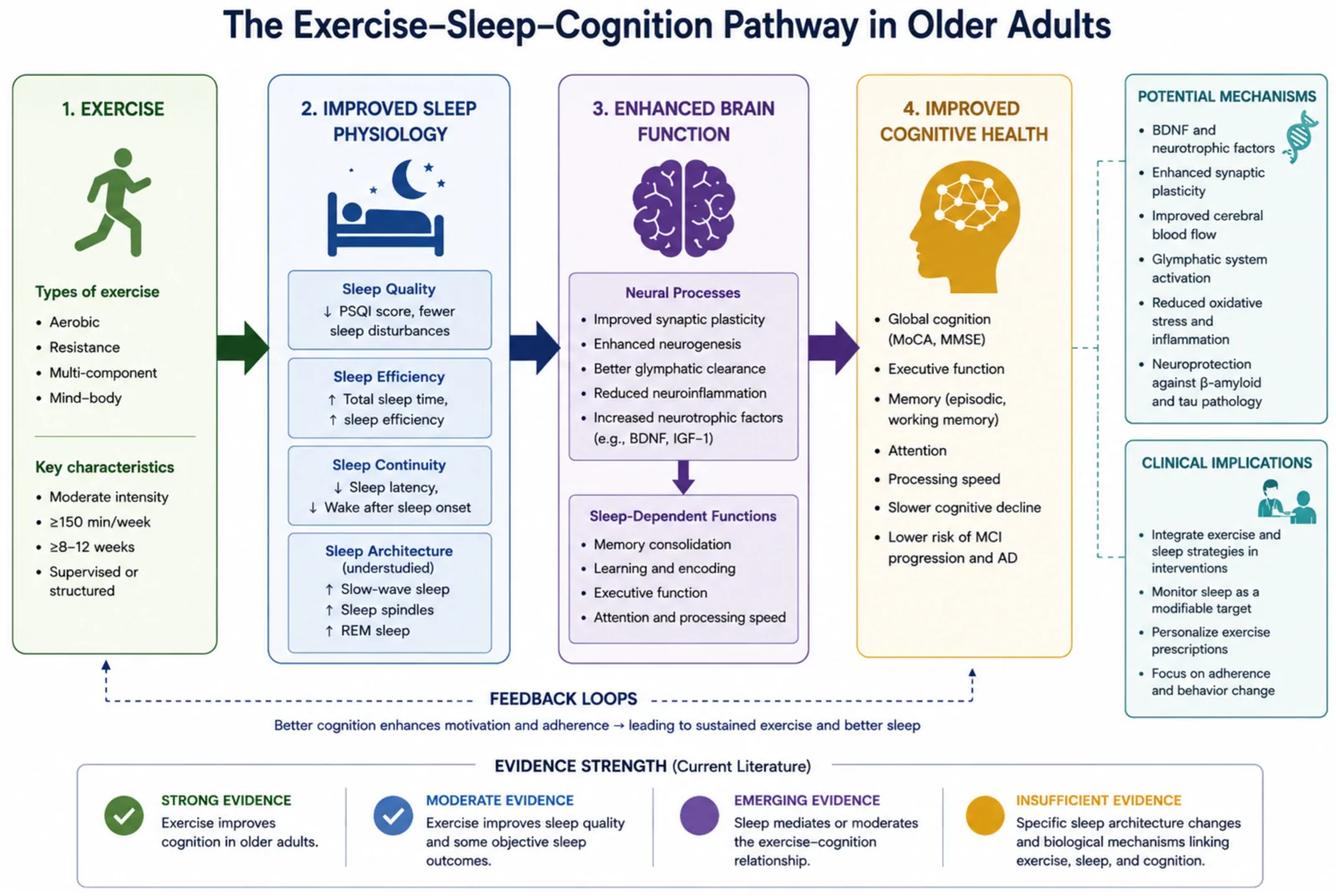
Conceptual framework illustrates the potential relationships among physical exercise, sleep physiology, brain function, and cognitive health in older adults. Exercise may influence cognitive outcomes directly through neurovascular, metabolic, inflammatory, and neurotrophic pathways and indirectly through changes in sleep. Sleep may act as a mediator of exercise-related cognitive benefits or as a moderator that influences the magnitude of cognitive responsiveness. Dashed pathways and feedback loops indicate relationships that remain uncertain or may be bidirectional. Created with BioRender.com by Vieira et al. (2026).

Although evidence linking physical exercise, sleep, and cognition has grown substantially, findings remain heterogeneous and have rarely been synthesized within a single conceptual framework [1,4]. Previous reviews have generally evaluated exercise–cognition and exercise–sleep relationships separately or have focused on specific interventions or clinical populations. In contrast, the present review synthesizes across intervention and observational studies to evaluate the relationships among physical exercise, sleep parameters, and cognitive performance in older adults, with particular emphasis on whether sleep may act as a mediator or moderator of exercise-related cognitive benefits (Figure 1). By identifying areas of convergence, inconsistency, and remaining knowledge gaps, this review also aims to inform future mechanistic and intervention research in cognitive aging.

## 2. Materials and Methods

This review was conducted as a structured integrative literature review, a methodological approach that allows the synthesis of evidence from diverse study designs, including experimental and observational research. The review followed established methodological guidance for integrative reviews [13].

Eligibility criteria were defined according to the Population, Intervention, Comparison, Outcome, and Study Design (PICOS) framework. Population (P): adults aged ≥60 years, including community-dwelling older adults and individuals with mild cognitive impairment (MCI). Intervention/Exposure (I): structured physical exercise interventions (e.g., aerobic, multicomponent, dance-based, Tai Chi, or Qigong) and/or habitual physical activity. Comparison (C): usual care, sedentary controls, alternative interventions, or differences in habitual physical activity or sleep characteristics in observational studies. Outcomes (O): cognitive performance, including global cognition (e.g., Montreal Cognitive Assessment [MoCA] and Mini-Mental State Examination [MMSE]) and domain-specific measures such as executive function, episodic memory, and processing speed. Study Design (S): randomized and non-randomized controlled trials, cohort studies, and observational studies.

Review articles, editorials, conference abstracts, theses, monographs, studies without full-text availability, and studies that did not concurrently evaluate physical activity or physical exercise, sleep, and cognitive outcomes were excluded. The timeframe was selected to capture the substantial growth in research evaluating the interrelationships among physical activity, sleep, and cognition in older adults. Although previous reviews have discussed these associations [14], emerging evidence from recent observational studies, neuroimaging investigations, and exercise intervention trials have considerably expanded understanding of the potential mechanisms linking physical activity, sleep, and cognitive health. Therefore, an updated synthesis of the literature was warranted to integrate findings published during the past decade and provide a contemporary overview of this rapidly evolving field.

Literature search was conducted from January 2015 to November 2025 across the following electronic databases: PubMed (Via MEDLINE), EMBASE, Cochrane Library, Scopus, and Web of Science. Google Scholar was additionally searched to screen potentially relevant grey literature. Controlled vocabulary terms (i.e., Medical Subject Headings) and free-text keywords were combined using Boolean operators (“AND” “OR”). The search strategy incorporated variations of the following terms: “older adults,” “aged,” “elderly,” “physical exercise,” “physical activity,” “sleep,” “sleep quality,” “sleep efficiency,” “cognition,” “cognitive function,” and “cognitive aging.”. No language restrictions were applied. Articles published in languages other than English were translated using DeepL/Google Translate for eligibility assessment and data extraction when necessary.

Study selection and data extraction were conducted independently by two reviewers (EFFSV and RLO). Titles and abstracts were screened independently, followed by full-text assessment of potentially eligible studies. Any discrepancies at any stage of the review process were resolved through discussion, and when necessary, consultation with a third reviewer (CG).

The study selection process followed four stages consistent with PRISMA recommendations: identification, screening, eligibility, and inclusion [15]. Reporting was conducted in accordance with the PRISMA 2020 statement. Titles and abstracts were initially reviewed to determine relevance to the research question. Duplicate records were identified and removed prior to screening. Potentially eligible studies were then assessed through full-text review to confirm compliance with inclusion criteria.

Data extraction was also performed independently by the same two reviewers using a standardized form (EFFSV and RLO). Data extraction was conducted using a standardized form developed for this review. The following information was recorded for each study: year of publication, country, study design, sample characteristics (including age and clinical status), description of the exercise intervention (i.e., modality, intensity, duration, and frequency), sleep assessment methods (e.g., subjective questionnaires, actigraphy and polysomnography) and sleep variables (e.g., sleep duration, sleep efficiency, sleep latency, total sleep time), cognitive domains evaluated, and principal findings. When reported, proposed physiological or neurobiological mechanisms were also documented.

Two reviewers (EFFSV and RLO) independently assessed the methodological quality and risk of bias of each included study using the Joanna Briggs Institute critical appraisal checklist appropriate to the study design (available in the OSF repository). Randomized controlled trials, quasi-experimental studies, and analytical cross-sectional studies were evaluated with their respective design-specific checklists. Each item was rated as “Yes”, “No”, “Unclear”, or “Not applicable”. Disagreements were resolved through discussion and, when necessary, consultation with a third reviewer (CG). Risk-of-bias assessments were used to inform interpretation of the evidence and were not used as an automatic basis for study exclusion.

The review methods were developed before completion of study selection; however, the protocol was not prospectively registered in PROSPERO or another formal systematic-review registry. Review materials and supporting data were subsequently deposited in the Open Science Framework (OSF). The absence of prospective protocol registration is acknowledged as a limitation because it reduces the ability to verify that all methodological decisions were specified in advance.

## 3. Results

**Figure 2:**
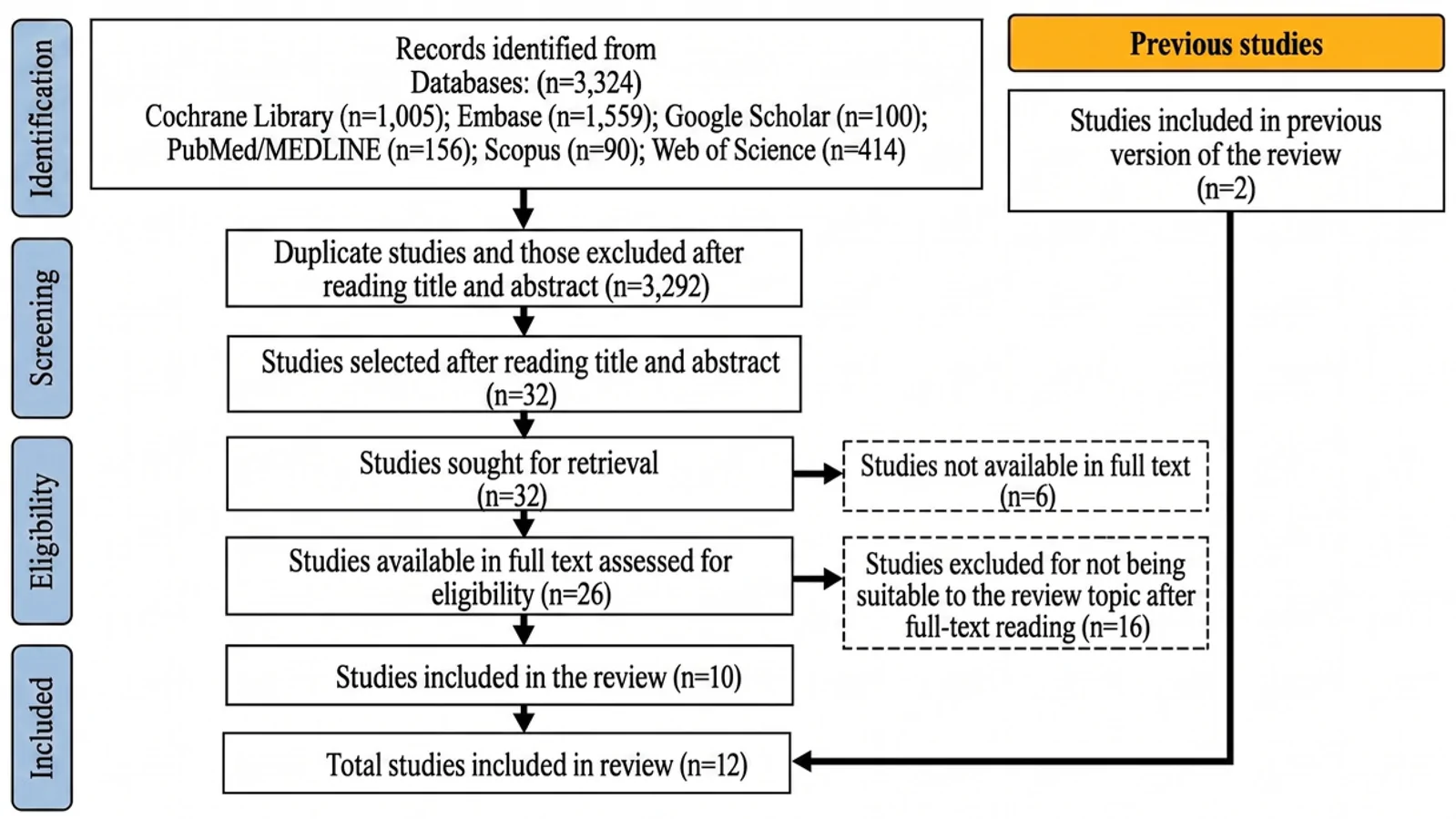
PRISMA flow diagram. Note: Adapted from [15].

The searches identified 3324 records. Following the removal of duplicate records and title-and-abstract screening, 32 reports were selected and sought for retrieval. Of these, six reports could not be retrieved, leaving 26 full-text reports that were assessed for eligibility. Sixteen full-text reports were excluded because they did not meet the review criteria. Ten studies identified through the database searches were included in the review, and two additional eligible studies were identified from the previous version of the review, resulting in a total of 12 studies in qualitative synthesis (Figure 2).

### 3.1. Population

The included studies represented diverse older adult populations. One trial focused on individuals with Parkinson’s disease [16]. Three studies specifically enrolled individuals with MCI [17,18,19]. Other studies included community-dwelling or cognitively unimpaired older adults [20,21,22,23]. One additional randomized trial enrolled older adults with sleep disorders and MCI [24]. The remaining studies involved broader community-dwelling older adult populations [25,26,27]. All observational studies included in the review employed a cross-sectional design. Overall, the included studies represented a diverse spectrum of older adult populations, ranging from cognitively healthy community-dwelling individuals to participants with MCI and Parkinson’s disease (PD). Although participant characteristics varied considerably across studies, this diversity strengthens the generalizability of the overall findings while also contributing to the observed heterogeneity in reported effects.

### 3.2. Intervention/Exposure and Comparison

Across the included studies, exercise interventions were predominantly structured and supervised, including aerobic, mind–body, and multicomponent exercise modalities [16,17,18,19,21,22,23,24,25]. Intervention duration ranged from 6 weeks to 6 months, with most programs lasting between 10 and 24 weeks [16,17,18,19,21,22,23,24,25]. Exercise sessions were generally performed two to five times per week and ranged from 12 to 60 min in duration, although sessions lasting 45–60 min were most common [16,17,18,19,21,22,23,24,25]. When reported, exercise intensity was generally moderate or moderate-to-vigorous and was prescribed using Borg ratings of perceived exertion, percentages of aerobic capacity, heart-rate reserve, or standardized movement protocols [16,17,18,19,22,24,25]. In observational studies, habitual physical activity, exercise frequency, or aerobic fitness were evaluated as exposure variables rather than structured interventions [20,26,27]. Despite differences in exercise modality, intervention duration, and study design, several common patterns emerged. Most interventions involved moderate-intensity exercise performed two to five times per week over approximately 10–24 weeks. Across studies, aerobic, multicomponent, and mind–body exercise programs consistently demonstrated favorable effects on sleep and cognitive outcomes, although the magnitude of these effects varied according to participant characteristics and intervention design.

A subset of studies implemented structured moderate-intensity exercise interventions lasting approximately 16–24 weeks [17,18,19]. These programs commonly involved sessions of approximately 60 min performed three times per week and were applied primarily in older adults with MCI [17,18,19]. Modalities included aerobic dance, step-aerobic exercise, and structured limb exercise. 

A second group of studies examined mind–body interventions, including Tai Chi, Qigong, and Yijinjing-based exercise [16,21,24]. These approaches typically combined low-to-moderate intensity physical movement with controlled breathing and attentional components. Intervention durations ranged from 6 to 10 weeks in some trials, although longer protocols were also observed [16,21,24]. These modalities were often applied in clinical populations, including individuals with mild cognitive impairment or Parkinson’s disease [16,24], and differed from conventional aerobic training in both movement structure and underlying behavioral components.

Other studies included multicomponent exercise approaches combining aerobic, strength, flexibility, and balance elements [23], as well as supervised aerobic cycling interventions delivered at moderate or high intensity [22]. These interventions were generally delivered two to three times per week and varied in duration, with some extending up to six months [22]. Observational studies within the included sample examined habitual physical activity or aerobic fitness as exposure variables rather than structured interventions [20,26,27]. Despite methodological differences, these studies contribute to the overall characterization of exercise behaviors relevant to older populations.

Information regarding the timing of exercise sessions was inconsistently reported across the included studies. Two mind–body intervention studies provided time-of-day information: the Baduanjin Qigong intervention was conducted at 9:00 a.m. [21], while Tai Chi Chuan sessions were conducted every morning [24]. However, the remaining structured exercise studies did not report the time of day at which sessions were performed, and none of the included studies explicitly described exercise timing in relation to participants’ habitual bedtime or sleep onset. Therefore, although available evidence suggests that some mind–body protocols were delivered in the morning, the potential influence of exercise timing relative to bedtime on sleep and cognitive outcomes could not be evaluated in the present review.

Control conditions across interventional studies typically included usual care, health education programs, or non-exercise comparators [16,17,18,19,21,24], allowing for evaluation of structured exercise effects relative to standard or inactive conditions.

### 3.3. Outcomes

The Pittsburgh Sleep Quality Index (PSQI) is a 19-item self-report instrument that evaluates sleep quality and sleep disturbances during the preceding month. It generates seven component scores: subjective sleep quality, sleep latency, sleep duration, habitual sleep efficiency, sleep disturbances, use of sleep medication, and daytime dysfunction. Each component is scored from 0 to 3, and the component scores are summed to produce a global score ranging from 0 to 21. Higher scores indicate poorer perceived sleep quality, and a global score greater than 5 is commonly used to identify substantial sleep difficulties [28]. Because the global score integrates multiple sleep dimensions, a one-point change cannot be interpreted as a fixed change in sleep duration or as a specific number of additional hours slept.

Sleep parameters were assessed using both objective and subjective methods, most commonly the PSQI, either as a global score or through subcomponents such as sleep latency, duration, and efficiency [16,17,18,19,21,22,24,26]. One trial incorporated objective actigraphy-derived indices including sleep efficiency and wake after sleep onset [24]. Cognitive outcomes were assessed using both global and domain-specific measures. The MoCA was the most frequently employed instrument and was used in several intervention studies involving older adults with mild cognitive impairment [16,17,18,24]. Other studies evaluated global cognition using the MMSE [16,25,26], while additional cognitive domains included executive function, episodic memory, attention, processing speed, and quality of life measures related to cognitive functioning [22,24,25]. Through intervention studies, improvements in sleep quality were frequently accompanied by improvements or maintenance of cognitive performance, particularly in global cognition, executive function, memory, and attention.

Several intervention studies reported significant improvements or moderation effects in cognitive outcomes following exercise participation. In Song et al. (2024), aerobic dance improved both sleep quality, assessed by the PSQI (β = −1.74, 95% CI −3.41 to −0.08, *p* = 0.04), and global cognitive function, assessed by the MoCA (β = 1.64, 95% CI 0.43 to 2.86, *p* = 0.008) [17]. Similarly, Song and Yu (2019) reported that moderate intensity aerobic exercise improved cognitive function (β = 1.895, 95% CI 1.421 to 2.368, *p* < 0.001) and health-related quality of life (β = 0.605, 95% CI 0.295 to 0.914, *p* < 0.001), with sleep quality contributing to the exercise–cognition relationship [18]. Wang et al. (2020) found that structured exercise improved general cognitive function at 12 weeks (mean difference = 1.20, 95% CI 0.354 to 2.054, *p* = 0.006) and 24 weeks (mean difference = 1.59, 95% CI 0.722 to 2.458, *p* = 0.001), with depressive symptoms, sleep quality, and processing speed partially mediating the intervention effect [19]. Liu et al. (2025) reported that Tai Chi Chuan combined with active rTMS resulted in lower PSQI scores (between-group mean difference = −3.1, 95% CI −4.2 to −2.1, *p* < 0.001) and higher MoCA scores (between-group mean difference = 1.4, 95% CI 0.7 to 2.1, *p* < 0.001) compared with Tai Chi Chuan combined with sham rTMS [24]. Sewell et al. (2023) found that baseline sleep efficiency moderated the effect of moderate-intensity exercise on episodic memory and global cognition, with participants reporting poorer baseline sleep efficiency showing improvements in episodic memory after the intervention [22]. Cross-sectional investigations frequently relied on self-reported sleep quality ratings [20,26,27].

Sleep outcomes were predominantly assessed using the PSQI, including both global scores and specific components such as sleep latency, sleep duration, sleep efficiency, and wake after sleep onset [16,17,18,19,21,22,24,26]. Across intervention studies, exercise was generally associated with improved sleep quality. Song et al. (2024) reported a significant reduction in PSQI scores following a 16-week aerobic dance intervention (β = −1.74, 95% CI −3.41 to −0.08, *p* = 0.04) [17]. Similarly, Liu et al. (2025) found that participants receiving Tai Chi Chuan combined with active rTMS demonstrated lower PSQI scores than those receiving Tai Chi Chuan combined with sham rTMS (between-group mean difference = −3.1, 95% CI −4.2 to −2.1, *p* < 0.001) [24]. Beyond direct improvements in sleep quality, several studies identified sleep as an important mechanism linking exercise and cognition. Song and Yu (2019) reported that sleep quality contributed to the relationship between aerobic exercise and cognitive improvement, whereas Wang et al. (2020) identified sleep quality as a partial mediator of exercise-induced cognitive benefits [18,19]. Furthermore, Sewell et al. (2023) found that baseline sleep efficiency moderated exercise-related changes in episodic memory and global cognition, highlighting the importance of sleep characteristics in shaping cognitive responses to exercise interventions [22].

The reported PSQI changes should be interpreted as changes in multidimensional perceived sleep quality rather than as direct changes in hours of sleep. For example, a reduction in the global PSQI score may reflect improvement in one or more components, including subjective sleep quality, sleep latency, sleep duration, sleep efficiency, sleep disturbances, medication use, or daytime dysfunction. Therefore, a one-point reduction cannot be equated with one additional hour of sleep. Although the reported changes were statistically significant in some studies, their clinical relevance remains less certain because a universally accepted minimal clinically important difference has not been established specifically for older adults with mild cognitive impairment. Clinical interpretation should therefore consider baseline PSQI scores, whether participants crossed the conventional threshold for poor sleep, the magnitude of component-level changes, and the presence of corresponding improvements in daytime function or objective sleep measures.

Among the studies reporting component-level results, the most frequently improved PSQI dimensions were subjective sleep quality, sleep latency, sleep duration, and habitual sleep efficiency. For example, the aerobic dance intervention reported improvements in global sleep quality, sleep latency, sleep duration, and sleep efficiency. However, component-level findings were not reported consistently across all studies, limiting conclusions regarding which specific dimensions of sleep were most responsive to exercise. Sleep disturbance, sleep medication use, and daytime dysfunction were less consistently described, and the absence of reporting should not be interpreted as evidence that these domains did not change.

Taken together, qualitative synthesis indicates a tendency toward converging findings across studies, suggesting that exercise interventions (particularly those involving moderate-intensity aerobic or multicomponent formats) may be associated with improvements in sleep parameters, which could, in turn, relate to better cognitive performance.

### 3.4. Synthesis of Intervention Characteristics

The methodological appraisal identified variation in risk of bias across the included evidence. The randomized trials were generally strongest in reporting eligibility criteria, outcome measurement, and statistical analysis, but several studies provided limited information on allocation concealment, blinding, or completeness of follow-up. The non-randomized intervention and cross-sectional studies were more vulnerable to confounding, selection bias, and uncertainty regarding the temporal direction of associations. Because the evidence base included heterogeneous study designs and several studies relied on self-reported sleep measures, the findings should be interpreted as suggestive rather than definitive. Complete item-level assessments are presented in Supplementary Table S1.

### 3.5. Characteristics and Patterns of Exercise Interventions

Across the included studies, a pattern emerges in which moderate-intensity aerobic and multicomponent interventions of short-to-moderate duration (typically between 12 and 16 weeks) were most frequently employed. These interventions were generally delivered in structured formats, with consistent weekly frequency and session duration. Mind–body modalities such as Tai Chi and Qigong followed similar temporal structures but differed in movement characteristics and intensity profiles (Table 1). Overall, the studies demonstrate convergence in intervention design, particularly regarding moderate intensity, structured delivery, and durations exceeding six weeks, providing a basis for interpreting their findings within a common framework. This descriptive synthesis provides the basis for interpreting the findings presented in the subsequent discussion.

## 4. Discussion

### 4.1. Exercise and Sleep as Interdependent Targets in Cognitive Ageing

The studies included in this review consistently investigated the relationship between physical exercise, sleep parameters, and cognitive performance in older adults. Across both interventional and observational designs, sleep-related variables frequently appear within analytical models assessing the association between exercise and cognitive outcomes. In several studies, sleep quality or sleep efficiency were evaluated as potential mediating or moderating factors within the relationship between physical activity and cognitive outcomes [18,19,20,21,22,23,25,27]. However, the limited number of studies evaluating these pathways precludes firm conclusions regarding their mechanistic role.

In interventional studies involving older adults with mild cognitive impairment, structured aerobic, multicomponent, and mind–body exercise programs were generally associated with improvements in sleep quality and cognitive outcomes, although the magnitude and consistency of these effects varied across studies. For example, aerobic dance and moderate-intensity aerobic exercise improved both sleep quality and global cognitive performance [17,18], while Tai Chi Chuan and Qigong-based interventions demonstrated beneficial effects on sleep quality and cognitive measures in clinical populations [21,24]. These findings are broadly consistent with previous systematic reviews reporting beneficial effects of aerobic, multicomponent, and mind–body exercise on cognitive performance in older adults and persons with MCI. However, relatively few reviews have specifically considered sleep as a potential mediator or moderator of exercise-related cognitive benefits. By integrating evidence from both intervention and observational studies, the present review extends previous work by highlighting sleep quality, sleep efficiency, and related sleep characteristics as recurring factors associated with cognitive outcomes following physical activity. Although the available evidence does not establish causality, the consistent inclusion of sleep-related variables in mediation and moderation analyses across independent studies suggests that sleep warrants consideration as more than simply a parallel outcome of exercise interventions.

Across interventional trials and observational analyses, sleep quality consistently emerged as a relevant factor within the exercise–cognition interface, either as an intermediary pathway or as a modifier of response [18,19,20,21,22,23,25,27]. This pattern is clinically meaningful. Sleep disturbance is highly prevalent among older adults and is often considered a secondary complaint rather than a central therapeutic target. However, the reviewed studies suggest that sleep quality may play an active role in shaping cognitive outcomes associated with behavioral interventions. These findings suggest that interventions that improve sleep may enhance the cognitive benefits of exercise, even if sleep itself is not the sole mechanism through which exercise influences brain health.

In several randomized trials involving individuals with MCI, improvements in global cognition were statistically linked to concurrent improvements in sleep indices [17,18,19,24]. While these findings do not establish direct causation, they consistently position sleep as a non-negligible component in the behavioral management of early cognitive decline. Rather than interpreting exercise-induced cognitive benefits as purely neurovascular or metabolic in origin, our results encourage a broader behavioral framework in which restorative sleep processes may facilitate or consolidate training-related adaptations. Collectively, these findings support the hypothesis that sleep may represent an important behavioral pathway linking exercise and cognitive health, while underscoring the need for mechanistic studies capable of determining the extent to which these associations reflect causal relationships.

### 4.2. Implications for the Management of MCI

The clinical relevance of these findings is particularly apparent in populations diagnosed with MCI. Pharmacological options for slowing progression remain limited, and lifestyle interventions have gained prominence as complementary strategies. Several trials included in our review showed that structured aerobic or multicomponent exercise programs were associated with stabilization or improvement in global cognitive measures among individuals with MCI [17,18,19,24]. Importantly, sleep quality frequently emerges within analytical models as a significant explanatory factor.

This raises a clinically important possibility: the cognitive effects of exercise in MCI may be partially contingent upon improvements in sleep continuity, latency, or efficiency. In practice, this suggests that prescribing exercise without assessing sleep disturbance may overlook a relevant modifiable contributor. Screening tools such as the PSQI, although subjective, are brief, feasible, and provide valuable information regarding perceived sleep quality in outpatient settings. However, subjective measures may not fully capture alterations in sleep architecture or objectively measured sleep continuity. Conversely, objective measures such as actigraphy provide complementary information on sleep duration, efficiency, and fragmentation but may not reflect an individual’s perception of sleep quality. Therefore, combining subjective and objective sleep assessments may provide a more comprehensive evaluation of sleep and its relationship with cognitive outcomes in older adults [24]. Although objective assessments may not be feasible in all clinical settings, they may be particularly valuable in research studies seeking to clarify the mechanisms linking exercise, sleep, and cognitive outcomes.

The PSQI is responsive to change overtime and has frequently been used to evaluate longitudinal changes following behavioral and exercise interventions. Studies outside the present review have also reported reductions in global PSQI scores following exercise programs in older adults, suggesting that perceived sleep quality can improve over intervention periods. Nevertheless, longitudinal changes are not uniform across populations. Greater improvements may be observed among participants with poorer baseline sleep, whereas individuals with relatively favorable baseline scores may have limited opportunity for measurable improvement. In addition, changes in the global score may be driven by different combinations of component scores across studies. Consequently, longitudinal PSQI findings should be interpreted alongside baseline sleep status, component-level results, intervention duration, and, where available, objective sleep measures.

Moreover, the inclusion of mind–body modalities such as Qigong and Tai Chi expand the spectrum of clinically viable options [21,24]. These modalities may be particularly appropriate for individuals with balance limitations, frailty, or lower exercise tolerance. Their demonstrated associations with improved sleep parameters further strengthen their translational appeal.

### 4.3. Sleep as a Treatment Modifier Rather than a Passive Outcome

A noteworthy observation across the reviewed studies is that sleep often acted as a modifier of exercise responsiveness rather than merely as a secondary outcome. In one randomized trial, individuals presenting with lower baseline sleep efficiency experienced greater episodic memory improvement following moderate-intensity cycling [22]. In another study, sleep duration interacted with multicomponent training effects, suggesting additive or synergistic influences [23].

Observational evidence reinforces this interpretation. Higher aerobic fitness was associated with superior executive functioning particularly in participants reporting better sleep quality and lower body mass index [20]. Similarly, exercise frequency was indirectly associated with cognitive performance through pathways involving sleep and depressive symptoms [27]. These findings imply that sleep characteristics may influence the cognitive “return” on physical activity.

The direction of the observed moderation effects was not consistent across studies. In some analyses, poorer baseline sleep was associated with greater exercise-related cognitive improvement, whereas other findings suggested that better or recommended sleep supported a stronger cognitive response to exercise. These results may partly reflect differences in the distribution of baseline sleep characteristics across study populations. In samples enriched for poor sleepers, participants with greater baseline impairment may have had more opportunity for improvement, producing an apparently greater response among those with poorer sleep. By contrast, in samples with broader or more favorable sleep distributions, adequate sleep may have supported physiological recovery, attention during training, and sleep-dependent consolidation of cognitive adaptations.

These findings are therefore not necessarily mutually exclusive. Poor sleep may identify a subgroup with greater potential for improvement, whereas adequate sleep may provide conditions that facilitate the expression of exercise-related cognitive benefits. Differences in cognitive status, exercise modality, intervention dose, sleep measurement, cut-off values, sample size, and statistical modelling may also explain the divergent results. Furthermore, effects observed at the extremes of the sleep distribution may not generalize to participants with intermediate sleep quality. Future studies should report the full distribution of baseline sleep scores, examine potential non-linear associations, prespecify interaction analyses, and determine whether moderation differs according to specific sleep components rather than the global PSQI score alone.

From a clinical standpoint, this supports a more individualized model of behavioral intervention. Rather than applying uniform exercise prescriptions, clinicians might consider baseline sleep disturbance as one dimension of risk stratification. Older adults reporting fragmented or insufficient sleep may represent a subgroup with both higher vulnerability and potentially greater responsiveness to integrated exercise-based strategies. Whether tailoring exercise interventions according to baseline sleep characteristics results in greater cognitive benefits remains an important question for future research.

### 4.4. Behavioral and Neurocognitive Mechanisms

Mechanistic evidence within the included studies was limited, with few investigations incorporating direct neurophysiological or biological markers. Nevertheless, several analytical models included sleep quality, depressive symptoms, and cognitive processing variables within the same framework, suggesting that these domains may interact within broader behavioral and neurocognitive pathways [19,21,27]. However, very few studies have assessed biological or neurophysiological markers associated with neuroplasticity or neurodegeneration. Only one included study incorporated a neurophysiological indicator [16] evaluated cortical activation patterns using functional near-infrared spectroscopy in individuals with Parkinson’s disease undergoing Yijinjing exercise training. The authors reported patterns consistent with exercise-related neural adaptation, suggesting possible neuroplastic responses associated with improvements in sleep and cognitive performance. However, no studies measured molecular biomarkers such as BDNF, inflammatory markers, or biomarkers associated with neuro-degenerative processes (e.g., amyloid-β or tau proteins).

Physical exercise may support cognitive health in older adults through several interacting neurobiological and behavioral mechanisms. Exercise can promote cerebral perfusion, vascular function, metabolic regulation, and neuroplasticity. Neurotrophic signaling, particularly involving BDNF, may support synaptic plasticity, hippocampal function, learning, and memory. Exercise may also influence inflammatory and oxidative pathways that are relevant to ageing and neurodegeneration. However, these mechanisms were rarely measured directly in the studies included in this review, and their contribution to the observed cognitive outcomes therefore remains inferential.

Skeletal muscle may contribute to exercise-related brain adaptation through the production of contraction-responsive signaling molecules. These muscle-derived signals may influence systemic metabolism, vascular function, and inflammatory regulation, thereby contributing indirectly to brain health. Although skeletal muscle can express BDNF, its role as a circulating mediator of exercise-related cognitive effects remains uncertain. Consequently, the muscle–brain pathway should be interpreted as a plausible component of a broader network rather than as an established single mechanism.

Sleep may interact with these exercise-related processes through several pathways. Adequate sleep supports memory consolidation, synaptic regulation, emotional regulation, and neural recovery. Exercise-related improvements in sleep continuity or efficiency could therefore create conditions that favor the stabilization of cognitive adaptations. Conversely, fragmented or insufficient sleep may impair attention, executive function, metabolic regulation, and participation in exercise. This bidirectional relationship may partly explain why baseline sleep characteristics influenced cognitive responsiveness in some studies.

Vascular, neurotrophic, inflammatory, metabolic, and sleep-dependent mechanisms are unlikely to operate independently. For example, improved sleep may support neuroplasticity, while improved physical fitness may reduce inflammatory burden and enhance cerebral perfusion. Changes in mood and depressive symptoms may also influence both sleep and cognitive performance. The available evidence therefore supports an integrated model in which exercise and sleep interact across multiple levels rather than through a single linear pathway.

Direct mechanistic evidence remains limited. Most included studies relied on behavioral outcomes and subjective sleep measures, and only one incorporated a neurophysiological assessment. Future trials should combine objective sleep assessment with neuroimaging, vascular measures, BDNF and other neurotrophic markers, inflammatory biomarkers, and domain-specific cognitive testing. Such designs would help determine whether sleep acts as a mediator, moderator, parallel outcome, or independent contributor to exercise-related cognitive change.

### 4.5. Practical Considerations for Healthcare Systems

A relevant aspect of the reviewed evidence is its potential applicability to clinical and community settings. Most interventions involved moderate intensity aerobic or multicomponent programs delivered two to five times per week for durations ranging from six to twenty-four weeks [16,17,18,19,21,22,23,24,25]. These formats are implementable in community centers, outpatient rehabilitation clinics, and primary care-linked wellness programs.

An additional practical consideration relates to the timing of exercise sessions. Although the reviewed studies generally prescribed exercise frequency and duration, few explicitly reported the time of day at which interventions were delivered. Emerging sleep research suggests that morning or early afternoon physical activity may support circadian alignment and improve nocturnal sleep quality, whereas vigorous exercise performed close to habitual bedtime may delay sleep onset in some individuals. For older adults experiencing sleep fragmentation or delayed sleep timing, scheduling structured physical activity earlier in the day may therefore maximize both sleep and cognitive benefits. However, evidence regarding the optimal timing of exercise for maximizing cognitive and sleep-related benefits remains limited and warrants further investigation.

Intervention adherence was not reported consistently enough to permit a reliable cross-study comparison. Where attendance or completion data were available, higher exposure to the prescribed exercise dose may have contributed to more favorable sleep or cognitive outcomes; conversely, low attendance, attrition, or reduced compliance with unsupervised sessions may have attenuated intervention effects. Adherence is also likely to differ by modality, supervision, physical limitation, transportation burden, and baseline sleep disturbance. Because adherence can influence both internal validity and real-world effectiveness, future trials should report attendance, achieved exercise dose, reasons for missed sessions, retention, and adherence to unsupervised components using standardized definitions. Intervention fidelity should likewise be monitored to distinguish limited treatment efficacy from incomplete intervention delivery. Additionally, group-based modalities such as dance programs may confer social engagement benefits, which themselves are relevant to cognitive health [27]. Beyond participant adherence, intervention fidelity, or the extent to which an intervention is delivered as intended according to the study protocol, should be considered when designing and interpreting exercise interventions [29]. Variability in how interventions are delivered may influence observed treatment effects and limit reproducibility, highlighting the importance of monitoring intervention delivery alongside participant participation and adherence [29].

Clinically, these findings support the integration of structured exercise into multidomain strategies aimed at preserving cognitive health in older adults. However, future interventions should simultaneously optimize exercise prescription, objectively monitor sleep physiology, assess intervention fidelity, and incorporate mechanistic biomarkers to better understand the pathways linking exercise, sleep, and cognition.

From a public health perspective, exercise interventions may offer scalable, low-cost strategies to address two prevalent concerns in ageing populations: sleep disturbance and cognitive decline. However, clinical translation requires careful consideration of comorbidities, physical limitations, individualized tolerance, and broader sleep hygiene practices. Interventions that combine physical activity with behavioral sleep recommendations (e.g., maintaining regular sleep schedules, limiting late-evening light exposure, reducing caffeine intake, and promoting daytime light exposure) may further enhance both sleep quality and cognitive outcomes in older adults. Individual differences in chronotype may further influence the effectiveness of exercise interventions. Older adults typically exhibit a shift toward earlier circadian timing (“morning-ness”), yet variability remains substantial. Exercise performed at times that align with an individual’s preferred circadian phase may enhance adherence, optimize alertness during training, and potentially strengthen downstream cognitive and sleep-related outcomes. Future interventions should therefore consider circadian preference when scheduling exercise sessions. From a clinical perspective, incorporating brief sleep assessments alongside exercise prescription may improve identification of individuals more likely to benefit from behavioral interventions.

Clinicians and rehabilitation professionals should screen for sleep disturbance when prescribing exercise to older adults, particularly those with mild cognitive impairment, and should select safe, progressive programs that reflect mobility, balance, cardiovascular risk, and patient preference. Healthcare services should consider integrating brief sleep assessment, exercise counselling, and referral pathways within primary care, geriatric, neurology, sleep, and rehabilitation settings. Community providers should prioritize accessible moderate-intensity aerobic, multicomponent, or mind–body programs with qualified supervision and systematic monitoring of attendance, adverse events, and achieved exercise dose. Policymakers should support low-cost community and remotely delivered programs, reduce transportation and digital-access barriers, and fund trials that include objective sleep assessment, longer follow-up, and clinically meaningful cognitive outcomes. These recommendations should be implemented as provisional practice considerations rather than definitive clinical guidelines because the current evidence base remains heterogeneous and does not establish an optimal modality, dose, or delivery format.

### 4.6. Limitations of the Evidence Base

Despite converging trends, the current literature presents important limitations. Heterogeneity across interventions complicates direct comparison, as exercise intensity, modality, and supervision varied considerably. In addition, sleep was assessed predominantly by using subjective questionnaires, such as the Pittsburgh Sleep Quality Index, with only limited use of objective measures such as polysomnography (PSG) or actigraphy [24]. Consequently, relatively little is known about whether specific aspects of sleep architecture, including slow-wave sleep, sleep spindles, or sleep-dependent oscillatory activity, contribute to exercise-related cognitive benefits. Future studies incorporating polysomnography (PSG) or wearable electroencephalography (EEG) may therefore provide important mechanistic insights.

Cognitive outcomes were frequently measured using global screening tools, which may lack sensitivity to subtle domain-specific change. Intervention durations were generally short to moderate, and long-term sustainability remains unclear. Additionally, cultural concentration of several studies may limit external validity. As this review employed qualitative synthesis rather than quantitative pooling, effect magnitude cannot be precisely estimated. Publication bias and small-study effects cannot be excluded.

Additionally, variability in sleep assessment methods and reliance on subjective measures may have influenced the consistency and comparability of reported findings across studies. These considerations highlight the need for integrative study designs that simultaneously account for behavioral, physiological, and cognitive domains when examining aging-related outcomes. An important observation across the included studies is that relatively few interventions specifically recruited participants with clinically diagnosed sleep disorders such as chronic insomnia or obstructive sleep apnea. Most studies evaluated subjective sleep quality using instruments such as the Pittsburgh Sleep Quality Index or included participants with self-reported poor sleep, rather than confirmed sleep pathology. Consequently, although current evidence supports sleep as a relevant mediator or moderator of exercise-related cognitive benefits, the generalizability of these findings to clinical sleep populations remains uncertain.

Future randomized trials should deliberately include participants with diagnosed sleep disorders and incorporate objective sleep assessments, such as polysomnography or actigraphy, to determine whether exercise-induced cognitive improvements differ according to sleep disorder subtype, severity, and treatment status. Although the available evidence is encouraging, the predominance of studies involving community-dwelling older adults and individuals with mild cognitive impairment highlights the need for future investigations in populations with clinically diagnosed sleep disorders, where the interaction between exercise, sleep regulation, and cognition may differ substantially. In addition, the review protocol was not prospectively registered, which limits transparency regarding whether all eligibility, appraisal, and synthesis decisions were established before the review was conducted.

### 4.7. Research Agenda

Current evidence supports an interaction between exercise, sleep, and cognitive performance in older adults, but several priorities remain before these findings can be translated into routine clinical practice:

- Conduct adequately powered randomized controlled trials with longer follow-up periods to determine the durability of exercise-induced cognitive benefits.
- Include participants with clinically diagnosed sleep disorders (e.g., insomnia, obstructive sleep apnea, rapid eye movement (REM) sleep behavior disorder) to improve clinical applicability.
- Standardize exercise prescription, including modality, intensity, frequency, and duration, to facilitate comparisons across studies.
- Combine subjective sleep questionnaires with objective assessments such as actigraphy and polysomnography.
- Incorporate mechanistic biomarkers, including neuroimaging, BDNF, inflammatory markers, and measures of glymphatic function, to clarify biological pathways linking exercise, sleep, and cognition.
- Evaluate whether improvements in sleep mediate clinically meaningful reductions in cognitive decline and progression from mild cognitive impairment to dementia.

## 5. Conclusions

The available evidence suggests that sleep may be relevant to the association between physical exercise and cognitive performance in older adults. Across a small and heterogeneous body of intervention and observational research, sleep quality, sleep efficiency, and sleep duration were examined as potential intermediary pathways or modifiers of cognitive response. However, the evidence does not establish causality, and the relative contribution of sleep to exercise-related cognitive outcomes remains uncertain.

Structured aerobic, multicomponent, and mind–body exercise programs may be feasible approaches for supporting sleep and cognitive health in selected older adults, including some individuals with mild cognitive impairment. Nevertheless, differences in study design, risk of bias, intervention characteristics, adherence reporting, sleep assessment, and cognitive outcomes preclude recommendations regarding an optimal exercise modality or dose.

The principal unresolved questions concern whether improving sleep directly enhances exercise-related cognitive benefits, which sleep characteristics are most influential, and whether the relationship differs according to cognitive status, sleep disorder, baseline sleep quality, exercise modality, or intervention adherence.

Future adequately powered randomized controlled trials should use standardized exercise reporting, objective and subjective sleep measures, prespecified mediation and moderation analyses, longer follow-up, and mechanistic biomarkers. Until such evidence is available, sleep assessment and exercise prescription may reasonably be considered complementary components of individualized healthy-ageing strategies, but the present findings should be interpreted as preliminary.

From an applied perspective, the findings support consideration of sleep assessment alongside individualized exercise prescription for older adults, particularly those with mild cognitive impairment or sleep disturbance. Integrated exercise and sleep-focused strategies may have potential in clinical, rehabilitation, community, and remotely delivered programs. Nevertheless, these applications should be regarded as promising and provisional until stronger mechanistic and longitudinal evidence becomes available.

## Figures and Tables

**Table 1:** Characteristics of studies included in qualitative synthesis.

Author	Objective	Study Design	Sample Characteristics	Intervention Details	Cognitive and Sleep Assessment Tools	Main Sleep and Cognitive Findings
Liu et al. (2025) [21]	Moderating and mediating roles of sleep in the effects of Qigong intervention on cognition	Non-randomized controlled trial	78 community-dwelling Chinese older adults (mean age 77.9 years); 70.2% classified as poor sleepers.	Qigong Baduanjin 1 session/day, 5×/week, 12 min each, for 10 weeks	PSQI; subjective cognition questionnaire (M3); objective cognitive performance (d′ recognition memory task).	Qigong improved subjective and objective cognition, particularly in participants with poor baseline sleep. Improvements in sleep quality mediated gains in subjective cognition, while baseline sleep moderated intervention effects.
Liu et al. (2025) [24]	rTMS on the right dorsolateral prefrontal cortex enhances benefits of Tai Chi Chuan on sleep and cognition	RCT	110 older adults (60–75 years) with sleep disorders and mild cognitive impairment (MCI).	Tai Chi Chuan (24 forms), 60 min, 5×/week, 6 weeks + active or sham rTMS (20 min, 1200 pulses, 5×/week, 6 weeks)	PSQI; actigraphy; MoCA; memory and executive function tests.	Tai Chi combined with rTMS significantly improved subjective and objective sleep quality, global cognition, memory, and executive function compared with sham stimulation.
Hu et al. (2025) [16]	Yijinjing-inspired exercises on sleep disorders in Parkinson using fNIRS	Three-arm controlled trial	96 persons with Parkinson’s disease and sleep disorders.	Yijinjing-inspired exercises; 8 weeks; 30 min/day; 5 days/week; moderate intensity (standardized postures, controlled breathing). Each session: 5 min warm-up → 20 min movement → 5 min recovery	PSQI; MoCA; MMSE; UPDRS; PDQ-39; fNIRS.	Yijinjing exercise significantly improved sleep quality and induced modest cognitive gains while promoting functional brain reorganization, supporting exercise-related neuroplasticity in Parkinson’s disease.
Song et al. (2024) [17]	Aerobic dance program on sleep quality in older adults with MCI and poor sleep	RCT	89 community-dwelling older adults (>60 years) with mild cognitive impairment and poor sleep.	Aerobic dance program; 3×/week; 60 min/session; 16 weeks; moderate intensity (Borg 12–14)	PSQI; MoCA.	Aerobic dancing significantly improved global sleep quality, sleep latency, sleep duration, sleep efficiency, and cognitive function in older adults with mild cognitive impairment and poor sleep.
Sewell et al. (2023) [22]	6-month exercise intervention on sleep, assessed pre- and post-intervention, and whether baseline sleep measures moderate exercise-induced cognitive changes	RCT	89 cognitively unimpaired community-dwelling older adults (60–80 years).	Ergometer cycling; 6 months; 2×/week; 50 min; moderate (50–60% VO_2_, Borg 13); intense (intervals 80% VO_2_, Borg 18)	PSQI (sleep efficiency, duration, sleep latency); comprehensive neuropsychological battery.	Exercise did not improve sleep measures. However, poorer baseline sleep efficiency predicted greater exercise-induced improvements in episodic memory and global cognition, indicating a moderating effect of sleep.
Hu et al. (2021) [26]	Association between sleep characteristics and cognitive function, as well as demographic data and living habits which potentially impact sleep in the elderly people	Cross-sectional study	1287 Chinese older adults (mean age 70.8 years) recruited from community settings.	No exercise intervention; observational study	PSQI; MMSE; MoCA; Clinical Dementia Rating (CDR).	Better sleep quality, longer sleep duration, higher sleep efficiency, and lower hypnotic medication use were associated with better cognitive performance. Regular exercise was associated with better sleep quality.
Vogel et al. (2021) [23]	Multicomponent exercise intervention in older adults, stratified by sleep duration	RCT	49 community-dwelling older adults (mean age 82.9 years).	Multicomponent exercise: 2×/week, 45 min, 16 weeks (warm-up, walking tasks, sitting gymnastics, flexibility, relaxation)	Sleep duration questionnaire; MoCA; Functional Reach Test; Chair-Rising Test; gait assessment.	Multimodal exercise improved cognitive capacity only among participants with recommended sleep duration, suggesting adequate sleep enhanced cognitive responsiveness to exercise.
Fausto et al. (2022) [25]	Moderating influence of baseline sleep quality and BMI on the cognitive and mood outcomes of a community-based cardio-dance exercise program among older African Americans	cohort study	64 older African American adults participating in a community-based exercise program.	Cardio-dance; 60 min/session; 5 months (40 sessions); intensity 65–80% HR reserve; Borg RPE 4–6; popular/Latin music; hip hop, merengue, samba, cumbia, salsa steps	Sleep quality questionnaire; MMSE; Digit Span; Trail Making Test; depression assessment.	Cardio-dance exercise improved attention and depressive symptoms. Participants with poor baseline sleep demonstrated greater improvements in executive function and attention following exercise.
Wang et al. (2020) [19]	Structured exercise program on cognition and test mediation of depressive symptoms, sleep quality, and processing speed in exercise-induced cognitive benefits	RCT	116 community-dwelling older adults with mild cognitive impairment (MCI).	Structured exercise program, which includes a 10 min limbering-up exercise, 40 min of upper and lower limbs exercise, followed by a 10 min relaxation exercise; 24 weeks, 3 sessions/week, 60 min each (12 weeks supervised + 12 weeks unsupervised)	PSQI; general cognitive function assessment; depressive symptoms; processing speed.	Structured limb exercise maintained cognitive function. Sleep quality, depressive symptoms, and processing speed jointly mediated exercise-related cognitive improvement, with sleep showing the strongest mediating effect.
Fausto and Gluck (2022) [20]	Aerobic fitness and cognition in African American older adults, and determine whether BMI and sleep quality moderated the relationship between aerobic fitness and cognition	Cross-sectional study	402 older African American adults (60–90 years).	Aerobic fitness measured by VO_2_max (6 min walk test)	Sleep quality questionnaire; neuropsychological battery assessing memory, executive function, attention, processing speed, language, and generalization.	Higher aerobic fitness was associated with better executive function. The relationship between fitness and hippocampal-dependent cognition was stronger in participants with better sleep quality and lower BMI.
Yuan et al. (2020) [27]	Multiple mediation roles of depression and sleep quality on the relationship between exercise frequency and cognitive function	Cross-sectional study	2469 community-dwelling older adults (≥60 years).	Weekly exercise frequency (0–7 days); lasting more than 20 min/day; intensity not specified	Sleep quality rating; MoCA; Geriatric Depression Scale (GDS).	Higher exercise frequency was associated with better cognition. Sleep quality indirectly contributed to cognitive performance through reduced depressive symptoms rather than acting as an independent mediator.
Song and Yu (2019) [18]	Moderate-intensity aerobic exercise program on cognitive function and health-related quality of life in Chinese older adults with MCI, and explore mediating roles of depressive mood and sleep quality in the exercise cognition relationship	RCT	120 community-dwelling Chinese older adults (>60 years) with mild cognitive impairment (MCI).	Group moderate-intensity step aerobic exercise; 16 weeks; 3 sessions/week; 60 min each; moderate intensity (Borg 12–14)	PSQI; MoCA; Geriatric Depression Scale (GDS); Quality of Life–Alzheimer’s Disease (QoL-AD).	Moderate-intensity aerobic exercise improved cognition and quality of life. Reduced depressive symptoms and improved sleep quality significantly mediated exercise-induced cognitive benefits.

**Abbreviations:** BMI, body mass index; CDR, Clinical Dementia Rating; fNIRS, functional near-infrared spectroscopy; GDS, Geriatric Depression Scale; MCI, mild cognitive impairment; MMSE, Mini-Mental State Examination; MoCA, Montreal Cognitive Assessment; PDQ-39, Parkinson’s Disease Questionnaire-39; PSQI, Pittsburgh Sleep Quality Index; QoL-AD, Quality of Life in Alzheimer’s Disease; rTMS, repetitive transcranial magnetic stimulation; UPDRS, Unified Parkinson’s Disease Rating Scale. **Note:** Sleep was assessed predominantly using subjective measures, most commonly the Pittsburgh Sleep Quality Index (PSQI), whereas one study also incorporated objective actigraphy-derived sleep measures. Cognitive assessments primarily included global cognitive screening instruments, such as the Montreal Cognitive Assessment (MoCA) and Mini-Mental State Examination (MMSE), with several studies additionally evaluating specific domains, including executive function, episodic memory, attention, processing speed, and quality of life. Intervention durations ranged from 6 to 24 weeks. Exercise intensity, when reported, was typically prescribed using the Borg Rating of Perceived Exertion (RPE) scale or percentage of heart rate reserve. Studies are presented according to their thematic contribution to the review, beginning with studies that directly examined sleep as a mediator or moderator of the exercise–cognition relationship, followed by studies addressing broader associations among exercise, sleep, and cognitive outcomes.

## Data Availability

The data supporting the findings of this study are publicly available in the Open Science Framework (OSF) repository at: https://osf.io/we6fu (accessed on 15 August 2026; DOI: https://doi.org/10.17605/OSF.IO/BRPWK).

## Supplementary Materials

The following supporting information can be downloaded at: https://www.mdpi.com/article/10.3390/medsci14050532/s1, Supplementary Table S1: Methodological quality assessment of the included studies using Joanna Briggs Institute critical appraisal checklists.

## Abbreviations

The following abbreviations are used in this manuscript:

AD	Alzheimer’s disease
ADRD	Alzheimer’s disease and related dementias
BDNF	Brain-derived neurotrophic factor
BMI	Body mass index
CDR	Clinical Dementia Rating
fNIRS	Functional near-infrared spectroscopy
GDS	Geriatric Depression Scale
HR	Heart rate
HRR	Heart rate reserve
MCI	Mild cognitive impairment
MMSE	Mini-Mental State Examination
MoCA	Montreal Cognitive Assessment
PDQ-39	Parkinson’s Disease Questionnaire-39
PSQI	Pittsburgh Sleep Quality Index
QoL-AD	Quality of Life in Alzheimer’s Disease
RCT	Randomized controlled trial
RPE	Rating of Perceived Exertion
rTMS	Repetitive transcranial magnetic stimulation
UPDRS	Unified Parkinson’s Disease Rating Scale
VO_2_	Oxygen uptake
VO_2_max	Maximal oxygen uptake
