# Supplementary material for: Exercise, Sleep, and Cognitive Performance in Older Adults: A Structured Review of Mechanisms and Clinical Implications
Source: Med Sci (Basel). 2026 Aug 31;14(5):532. doi: 10.3390/medsci14050532 (PMC13609376; doi:10.3390/medsci14050532)
Supplement: Supplementary file 1 [file medsci-14-00532-s001.zip › medsci-4447397-Supplementary.pdf]

Supplementary Table S1. Methodological quality assessment of the included studies using Joanna Briggs Institute critical appraisal checklists.

Study	Study design	JBI checklist	JBI criteria	Methodological concern
Wang et al., 2020	RCT	JBI RCT	9/13	Lack of blinding of intervention personnel
Vogel et al., 2021	RCT	JBI RCT	9/13	Attrition and mean substitution for missing data
Song et al., 2024	RCT	JBI RCT	10/13	Participant and interventionist blinding not feasible
Song & Yu, 2019	RCT	JBI RCT	11/13	Participant and interventionist blinding not feasible
Liu et al., 2025	Non- RCT	JBI Quasi-experimental	6/9	Residual confounding due to non-randomized allocation
Yuan et al., 2020	Analytical cross-sectional study	JBI Cross-sectional	5/8	Self-reported exposure and sleep measures; residual confounding
Sewell et al., 2023	Secondary analysis of a RCT	JBI RCT	9/13	Secondary exploratory analysis with exclusions for incomplete baseline data
Fausto et al., 2021	Non-randomized propensity-matched cohort study	JBI Quasi-experimental	6/9	Self-selection and no-contact control group
Hu et al., 2021	Analytical cross-sectional study	JBI Cross-sectional	7/8	Potential selection bias due to low participation rate
Hu et al., 2025	RCT	JBI RCT	9/13	Participants and therapists were not blinded
Liu et al., 2025	RCT	JBI RCT	11/13	Limited blinding inherent to the intervention
Fausto & Gluck, 2020	Analytical cross-sectional study	JBI Cross-sectional	7/8	Sleep quality assessed using a single self-report item

Note: Methodological quality was assessed using the Joanna Briggs Institute (JBI) critical appraisal tools appropriate to each study design. Randomized controlled trials, quasi-experimental studies, and analytical cross-sectional studies were evaluated using their respective JBI checklists. The "JBI criteria" column indicates the number of criteria rated "Yes" over the total number of applicable checklist items. Ratings were used to support interpretation of the evidence and were not applied as an automatic exclusion criterion. JBI, Joanna Briggs Institute; RCT, randomized controlled trial.
